# Baltic Adolescents’ Health Behaviour: An International Comparison

**DOI:** 10.3390/ijerph17228609

**Published:** 2020-11-19

**Authors:** Leila Oja, Agnė Slapšinskaitė, Jaanika Piksööt, Kastytis Šmigelskas

**Affiliations:** 1 National Institute for Health Development, 11619 Tallinn, Estonia; leila.oja@tai.ee; 2 Department of Sports Medicine, Faculty of Nursing, Medical Academy, Lithuanian University of Health Sciences, LT 44307 Kaunas, Lithuania; agne.slapsinskaite@lsmuni.lt; 3 Health Research Institute, Faculty of Public Health, Medical Academy, Lithuanian University of Health Sciences, LT 44307 Kaunas, Lithuania; kastytis.smigelskas@lsmuni.lt; 4 Department of Health Psychology, Faculty of Public Health, Medical Academy, Lithuanian University of Health Sciences, LT 44307 Kaunas, Lithuania

**Keywords:** physical activity, vegetables, soft drinks, BMI, Baltic countries, HBSC, adolescents

## Abstract

The aim of the study is to assess the time trends in Baltic adolescents’ physical activity, dietary habits and BMI and compare the results with the average of the Health Behaviour in School-aged Children (HBSC) study. The research used HBSC data from 2006 to 2018. The total number of respondents was 17,458 in Estonia, 18,416 in Latvia and 20,466 in Lithuania. A logistic regression analysis was applied to estimate time trends in health behaviour indicators. The results demonstrated that Baltic adolescents’ physical activity has declined over the study years, except for Lithuanian girls. The prevalence of overweight adolescents has significantly increased since 2006. Dietary habits improved in all three Baltic countries, as consumption of vegetables increased, and soft drink consumption decreased during this time period. This research shows that a nationwide, highly representative study with health behaviour indicators enables us to assess regional differences compared to the HBSC average. The prevalence of overweight and obese adolescents in the Baltic countries has increased and moved closer to the HBSC average. Although Baltic adolescents’ daily vegetable consumption has increased over the last decade, it is still lower than the HBSC average.

## 1. Introduction

In general, young people in the WHO European Region enjoy better health and development than ever before but they are still failing to achieve their full health potential [1]. Health experience during the critical period of adolescence has short- and long-term implications for individuals and society [1]. Many health and risk behaviours are adopted and established during adolescence years [2]. Often, healthy habits/behaviours continue into adulthood and affect the health status and mortality of the population [1]. Studies so far have confirmed that the prevalence of poor subjective health outcomes increases with age and afflicts girls more than boys, with the gender gap increasing with age [3]. There is substantial cross-national variation in the prevalence of subjective health outcomes, especially for self-rated health and multiple health complaints [3].

Adolescents have many common health problems regardless of their origin and residence country, but prevalence differs by country/region, age and gender [3]. In the regional comparisons, Estonia, Latvia and Lithuania are considered as a single Baltic region, but in particular, we still lack scientific knowledge in which indicators countries show similar trends and which they differ. So, even if Baltic countries have highly similar historical and social backgrounds [4], it is still important to evaluate the health behaviour of adolescents in the Baltic region as compared to other Health Behaviour in School-aged Children (HBSC) study countries. We lack an international report that clearly shows adolescents’ health dynamics among Baltic countries. Specifically, the health of Lithuanian adolescents compared to other EU countries [5], and physical activity in the Baltic youth [6] were thoroughly analysed over a decade ago. At the root of Baltic adolescent’s health, there is a visible shortage of reports depicting the behaviour. Consequently, this article fosters us to minimise this gap within the literature and to better understand the dynamics of the indicators in Baltic countries.

At the most general level, daily physical activity is essential for optimal physical growth and development of the child [7]. The literature shows that engaging in physical activity and spending less time in screen-based sedentary behaviours are related to fewer health complaints [8,9]. Physical activity has numerous positive effects on child and adolescent health, with good evidence for beneficial effects on adiposity in normal-weight and overweight children and adolescents [10]. Despite the vast amount of knowledge available that states that adolescents partaking in regular physical activity are more likely to adopt a healthy and balanced diet that helps to improve physical and personal well-being [11], adolescents’ physical activity has gradually decreased.

Similarly to physical activity, daily consumption of vegetables and soft drinks has been used as an indicator of a healthy lifestyle. Vegetable consumption among adolescents is linked to many positive health outcomes. Fruit and vegetable consumption is commonly considered as an indicator for overall diet quality, also previously described as weak modifiable behavioural determinants of overweight [12]. Previously it was demonstrated that lower family affluence tends to be associated with higher soft-drink consumption, however the reversed pattern is applicable for Eastern European countries and Baltic countries. Consumption in aforementioned countries additionally may be considered as an indicator of wealth. The lowest levels of fruit and vegetable consumption are found in Northern Europe and the Baltic countries [1].

Consumption of fatty foods and a high sugar diet, combined with low physical activity, are the main causative factors of childhood obesity. To prevent and decrease obesity, dietary changes and the introduction of physical activity are the most promising methods [13]. The body mass index (BMI) is the most common index of adiposity status among children and adolescents. The previous HBSC studies have shown that, in general, overweight and obesity decrease with increasing age and boys tend to have significantly higher prevalence in almost all countries and regions at all ages [1,3]. The 2013/2014 HBSC study, for instance, indicated that increased prevalence of overweight adolescents was associated with low family affluence for boys in around half of the countries/regions and about two thirds for girls [3]. High rates of overweight/obesity and a poor level of daily moderate-to-vigorous physical activity among children from low affluence families provide disturbing evidence highlighting the necessity of public health efforts to implement obesity reduction interventions for this disadvantaged population [14].

It is important to better understand the factors associated with health and well-being, and their interrelation with family affluence for better strategy development, with the purpose of increasing the health of adolescents [15]. The family affluence scale (FAS) is a valid measure for adolescent wealth, and a proxy of socioeconomic status [15], which has evolved to be one of the most common tools or standard measurements used to assess the objective socioeconomic status of adolescents within the HBSC study [16]. In this analysis, we address the health of adolescents in relation to the FAS, physical activity, vegetables and soft drinks intake and overweight prevalence.

Low relative affluence and poor subjective health emerges as a consistent significant association. Family wealth may have an indirect effect on health; however, other proximal determinants should be investigated [3]. Therefore, it is important to keep track of trends and changes that are specific to more prevalent health behaviour variables. In this paper we explain the changes in adolescents’ health behaviour in addition to the similarities and differences among Baltic countries, and more generally, compared to the HBSC average results. Consequently, our study aimed to assess trends in adolescents’ physical activity, eating behaviours and BMI, and their differences in Baltic countries from 2006 to 2018.

## 2. Materials and Methods

### 2.1. Sample

This study is based on the data from the Health Behaviour in School-aged Children (HBSC) survey. Estonia, Latvia and Lithuania are part of 50 countries and regions that make up the HBSC Network. The HBSC is a school-based survey of adolescent health behaviours, carried out every four years. Standardized HBSC questionnaires were used in nationally representative samples. More detailed information about the HBSC methodology can be found in Roberts et al. [17].

Table 1 presents the characteristics of the study sample. The sample size in the Baltic countries is quite similar in terms of survey years, age and gender. Data from the Baltic countries represent between 5% and 7% of the total sample of HBSC in different survey years.

The study was approved by the Tallinn Medical Research Ethics Committee (No. 2100, 12.10.2017), the Ethics Committee for Biomedical Research, Lithuanian University of Health Sciences (No. BEC-89, 08.03.2018) and the Committee of Ethics of the Medical and Biomedical Research, Riga East Clinical University Hospital (No. 11-A/17, 05.10.2017). Written informed consent was obtained from all school principals and teachers included in the study. Passive consent was used on the student and parental level. The students had the possibility to withdraw their participation at any time during the data collection, they could also leave out questions that they did not want to answer.

### 2.2. Measures

Pupils reported their gender, age, and school grade. In order to assess socioeconomic status, the family affluence scale (FAS) III was used [18], which consists of six items, including: parental car ownership (response options none, one, two or more); sharing or not sharing bedroom (Yes or No); number of holidays per year (none, once, twice, more than twice); having computers at home (none, one, two, more than two); bathrooms at home (none, one, two, more than two); and having a dishwasher (Yes or No). Based on the six items, a sum from 0 to 9 points was calculated and used as a covariate in the regression analysis.

For estimating the prevalence of overweight adolescents, the body mass index (BMI) was used. HBSC has adopted the international BMI standards for children and adolescents, based on the work of Cole and others [19] that are recommended by the International Obesity Task Force (IOTF). Respondents were asked how much they weighed without clothes and how tall they were without shoes. These data were recorded in centimetres and kilograms, respectively, to calculate the BMI ((weight (kg) divided by height (m^2^)). The age- and sex-specific IOTF cut-offs were used in order to separate overweight or obese respondents [20].

To assess moderate-to-vigorous physical activity, schoolchildren were requested to rate the number of days over the past week during which they were physically active for a total of at least 60 min per day. Answers were provided to indicate the number of days per week (from 0 to 7). Responses were dichotomized into everyday vs. less than daily, according to the physical activity guidelines by WHO [21].

For estimating eating habits, the intake of vegetables and soft drinks was estimated. Respondents were asked how many times a week do they usually eat vegetables or drink soft drinks. Soft drinks were defined as “Coke or other soft drinks that contain sugar”. Response options ranged from “never” to “more than once a day”. The findings presented here are the proportions that reported drinking soft drinks or, respectively, eating vegetables at least once a day.

### 2.3. Data Analysis

For statistical analyses, IBM Statistics SPSS version 22 software was used. Percentages and 95% confidence intervals (CI) were calculated in order to estimate differences between Baltic countries and HBSC total (Table 2). Binary logistic regression analysis was used in order to assess changes in adolescents’ health behaviour by survey years, using 2006 data as a reference category in all analyses (Table 3, Table 4, Table 5 and Table 6). Regression analysis was conducted in two stages. First, only survey year was included into the model, in order to obtain unadjusted odds ratios. At the next stage, the results were adjusted for age group (11-year olds as a reference category) and FAS, which was included as a covariate. Results were provided as odds ratios (OR) and 95% confidence intervals (CI). Values of *p* < 0.05 were considered statistically significant.

## 3. Results

The study was based on data from the 2006–2018 HBSC international survey and the data were extracted to give information about Estonia, Latvia and Lithuania. We examined trends in adolescent health behaviours in four domains—moderate-to-vigorous physical activity, vegetable and soft drinks intake and BMI in relation to age, gender and FAS.

Prevalence of the health-related variables by country, year and gender are presented in Table 2. Daily moderate-to-vigorous physical activity of Estonian (17.3% were active at least 60 min each day) and Lithuanian (20.4%) boys is significantly lower than the HBSC total (23.2%), as among Latvian boys the proportion is about the same level (22.2%). Among girls, only Estonian schoolchildren are significantly below HBSC average.

Consumption of vegetables is significantly lower in all Baltic countries as compared to the HBSC total, both among boys and girls (Table 2). Consumption of soft drinks, on the positive side, was found to be significantly lower in Baltics, except Lithuanian boys, whose soft drinks intake in 2018 is about the same level as the HBSC total.

The prevalence of overweight or obese adolescents in the Baltic countries has moved closer to the HBSC average in each survey year. Since 2018, the prevalence of overweight or obesity in Estonian boys and girls and Latvian girls has been higher than among the HBSC countries in total.

When comparing the study years, as well as the effect of FAS and age, we can see that the trend of moderate-to-vigorous physical activity among Baltic adolescents is negative between 2006 and 2018 (Table 3). The family affluence gradient is relevant for the physical activity of adolescents in the results of boys and girls in all Baltic countries. It is also apparent from the results that physical activity reduces significantly by age groups, both for boys and girls.

The daily consumption of vegetables has increased since 2006 (Table 4). The biggest changes have occurred among adolescents in Estonia, both for boys (OR = 1.74 (1.51–2.00)) and girls (OR = 1.88 (1.65–2.14)). After adjusting the results for age and FAS, there are significant changes among Estonian, Latvian and Lithuanian boys and Estonian and Lithuanian girls, but not among Latvian girls.

Unfortunately, the consumption of vegetables decreases by age among adolescents in Baltic countries (Table 4). FAS is positively related with vegetable consumption among boys and girls in all three Baltic countries. The highest FAS effect on the consumption of vegetables is in Estonia (OR = 1.11 in boys and 1.12 in girls).

Soft drinks consumption has generally decreased when compared to 2006 (Table 5). Only Lithuanian boys have remained at the same level of soft drinks consumption. When adjusting the results for age group and FAS, soft drinks consumption has declined significantly among Baltic adolescents in all three countries except for Lithuanian boys. The biggest decrease is among Latvian girls (OR = 0.30 (0.24–0.39)).

FAS is positively related to soft drinks consumption in boys in Estonia (OR = 1.10 (1.06–1.15)) and Latvia (OR = 1.07 (1.04–1.11)), while in other groups better family affluence does not affect adolescents’ soft drinks consumption (Table 5). In age comparison, soft drinks consumption in 11-, 13- and 15-year olds are similar. Only Latvian 13-year-old boys consume soft drinks 1.24 times more than 11-year olds. Estonian 15-year-old girls consume about half as many soft drinks as 11-year olds.

Overweight or obesity is almost two times higher among boys and three times higher among girls when compared to 2006 (Table 6). The smallest change in weight is in Estonian boys. Considering the impact of FAS on BMI, higher family affluence significantly reduces obesity in Estonian boys and girls and Lithuanian girls, but not in Latvian adolescents and Lithuanian boys.

## 4. Discussion

### 4.1. General Discussion

Physical activity and eating behaviours established during childhood and adolescence are likely to persist into adulthood. Further, adolescence is a vulnerable period for the development of obesity, as weight tracks into adulthood [22]. Consequently, achieving and maintaining a healthy body weight is vital for favourable health outcomes. Moreover, lifestyle behaviours may change with increased autonomy during adolescence, so this life stage is important for appropriate behaviour consolidation. This study contributes to the limited number of publications depicting the behaviour—in all Baltic countries—of adolescent eating, physical activity and BMI between years of 2006 and 2018.

The physical activity patterns of adolescents in Baltic countries over recent decades have been shifting away from an active, towards a more unhealthy inactive direction. According to our results, moderate-to-vigorous physical activity has declined over the years of the study, except for Lithuanian girls. An alarming sign towards the effectiveness of healthier behaviour interventions is present, as the number of adolescents achieving the recommended physical activity level has been decreasing year by year. Even though Latvian boys are more active than boys in other Baltic countries, their physical activity decrease is the largest compared to 2006. Adolescence was and still is a critical age in terms of adherence to physical activity [23]. 

On the positive side, this study found that in all three Baltic countries vegetables consumption has increased, and soft drink consumption has decreased between 2006 and 2018. Even though daily vegetable consumption has increased over the last decade, it remains below the HBSC average. More positive shifts were seen in the eating behaviours of Baltic adolescents. Specifically, the consumption of soft drinks has generally declined compared to 2006, with consumption below the HBSC average. The role of nutrition, as an important lifestyle factor, in the timing of puberty has been acknowledged [24]. The tendencies in nutrition among Baltic adolescents are healthier, however this is not reflected in BMI data.

Obesity in children aged 5–19 years in almost all European regions has increased rapidly from 1980 to 2016 [25]. According to our study years, obesity has almost doubled. The results among Baltic countries’ adolescents for health indicators demonstrate a decline in physical activity (except for Lithuanian girls), and an increase in overweight, even though dietary habits appear to have improved. Many features of adolescent behaviours and environments can be disruptors of energy balance. The multifactorial origin of overweight and obesity indicates that various factors relate to excessive fat gain. The recent rise in obesity prevalence is primarily caused by environmental changes affecting diet and activity levels; however, large variation in the prevalence of obesity within populations suggests that some children are more susceptible than others to the obesogenic environment [26]. Relationships between lifestyle behaviours and body weight elucidate the inconsistent associations in the weight status of adolescents. Important advice to fight the increasing trend in overweight and obesity among children is to use preventive programmes, as research carried out in this direction shows positive results [25]. According to Nittari and others [25], it is not enough to raise awareness among children, but it is necessary that they have the support of adults to effectively carry out healthy programmes regarding physical activity and a balanced diet.

Previous studies have demonstrated that not meeting the recommendations for moderate-to-vigorous physical activity was the only significant healthy weight behaviour associated with both overweight and obesity, and solely obesity [27]. In Baltic countries the number of overweight adolescents has significantly increased; Estonian boys and girls and Latvian girls have outperformed the HBSC average. Of specific interest herein, a decline in adolescent physical activity was present with an increase in obesity among both boys and girls. The number of overweight or obese adolescents in the Baltic countries has moved closer to the HBSC average in each survey year. Since 2018, Estonian and Lithuanian boys and Estonian and Latvian girls have exceeded the HBSC average for overweight or obese adolescents. At the same time, approximately 40% of children and adolescents in Greece are either overweight or obese, exhibiting high prevalence rates of obesity in Europe [28]. Considerable evidence suggests that physical activity is the strongest behavioural determinant of adiposity, also our findings somehow support the notion that lifestyle behaviours synergistically contribute to BMI status, and reinforce the need to study the relationships between these behaviours and weight status concurrently.

Many of the nutritional and physical activity recommendations are similar—independently—in different geographical, socioeconomic and cultural contexts. The trends in all three Baltic countries show that FAS is positively related with vegetable consumption and physical activity among boys and girls. However, the effect of FAS on adolescent obesity is not entirely clear. In the case of Estonian adolescents, FAS influenced obesity in boys and girls, in addition to Lithuanian girls, but not Lithuanian boys and not at all in Latvian adolescents. Estonian boys had the highest proportion of overweight and obese schoolchildren, but Lithuanian boys had the highest increase in obesity in 2006–2018. According to previous HBSC studies [3], boys and girls with a high FAS scale are more likely to achieve 60 min of moderate-to-vigorous physical activity daily in more than half of the countries and regions. The difference between high- and low-affluence groups was 10 percentage points or less in most, but the relationship between family affluence and soft-drink consumption was not consistent across countries and regions. Socioeconomic factors might influence the healthy weight connected behaviours in more complex ways.

### 4.2. Strengths and Limitations

A main strength of our study is its nationwide coverage with a large representative sample and exploration of concurrent engagement in healthy behaviour indicators in all Baltic countries. Another strength is the rather a long study period between the years 2006–2018, and the comparison with HBSC survey average. However, there may have been some important factors that might have influenced and impacted physical activity, dietary habits or BMI of adolescents but were left out of the scope of this study. Several limitations should be considered when interpreting study findings.

First, all responses in the HBSC study are self-reported, leading to potential recall bias. As regards the differences in self-reported versus the direct height and weight, physical activity measurements noted, self-reported values tend to be typically greater than objectively measured values [29], while the self-reported weight tends to be lower when measured for girls and higher for boys [30]. This suggests that the rates provided in this study may not estimate the true magnitude of the level of overweight and obesity together with physical activity, even though the behavioural trends within the Baltic countries are noted and kept throughout the year. Self-report surveys function as a useful method for the purposes of behavioural trend surveillance, but direct measures could help to capture these health-related behaviours more precisely.

### 4.3. Implications

So why target adolescence in Baltic countries altogether? All things considered, throughout the study years in this transitioning life stage, diet was generally shown to exhibit a healthier trend, however, physical activity was shown to be decreasing to ever more insufficient levels, while weight is shifting more towards overweight or obese. Hence, continued promotion of physical activity and healthy dietary patterns (Baltic countries are still below the HBSC average in consumption of vegetables), as well as prevention and control of childhood obesity, is of notable importance in this European region. Findings from this study could have implications towards policies and programmes targeted at reducing BMI and increasing the physical activity rates of adolescents. From the existing literature, maintenance of lifestyle approaches seems to be better in children than in adults, suggesting that it may be easier to motivate children and modify their behaviour [31].This may also be the case for adolescent’s physical activity levels, whose habits may not be as conditioned as in adulthood. Moreover, high BMI in adolescence is difficult to reverse and so the intention should be to prevent this from childhood, with special interventions in kindergartens and schools in all Baltic countries dealing with this problem together in the region. The emphasis on physical activity should be stronger once trying to demonstrate not only the health benefits, but also the exciting part of involvement in physical activity, in terms of the social support from family, school and peers associated with an active lifestyle.

## 5. Conclusions

As well as the cultural and historical–political background in the Baltic States being similar, the patterns of health behaviours of adolescents in Estonia, Latvia and Lithuania since 2006 are also quite similar. In general, it can be noted that while in 2006 the prevalence of overweight adolescents in the Baltic states was about one third lower than the HBSC average, by 2018 this indicator was even higher than the HBSC average. In 2018 the level obesity and overweight is almost twice as high in boys and three times as high among girls, when compared to 2006. The effect of FAS on BMI has not been unequivocally proven, as higher family wealth significantly reduces obesity among Estonian adolescents and Lithuanian girls, but not among Latvian adolescents. Consumption of soft drinks also follows the average trend of HBSC, as consumption is declining among both boys and girls in 2006–2018. Although the consumption of vegetables in the Baltic countries has increased in each year of the study, it is still below the HBSC average. In all Baltic countries the trend of moderate-to-vigorous physical activity among adolescents is negative between 2006 and 2018, but the results are relevantly influenced by the family affluence gradient. In terms of physical activity, Estonian adolescents should follow the example of their neighbours; in 2006–2018, more adolescents in Latvia and Lithuania have been physically active for at least 60 min every day than in Estonia.

Although the changes in Baltic adolescents’ health behaviours between 2006 and 2018 are quite similar, the study also enables us to highlight the differences between the three countries and address the most problematic topics for each country. When compared to their neighbours, Estonian adolescents’ lower physical activity together with higher overweight/obesity rates represent the topics that need more attention. Lithuania and Latvia, on the other hand, need to focus on improving adolescents’ dietary habits, specifically, on reducing soft drinks consumption in Lithuania and eating more vegetables in Latvia.

This study highlights the importance of exploring the long-term changes in health indicators, with a special focus on obesity, physical activity and nutritional habits. According to our findings, obesity rates in Baltic adolescents almost doubled, levels of physical activity diminished, while nutritional habits improved throughout the study years. Taking into account the lack of physical activity alongside the improvements in eating habits, obesity and overweight are still problems. This outcome further brings our attention to the energy balance between consumed and expended calories, which must be respected. Physical inactivity is especially concerning when viewed through a health lens as calories that are not expended are converted into fat, which further predisposes health problems connected with overweight and obesity.

## Figures and Tables

**Table 1:** Number of respondents by country, survey year, gender and age group.

Year	Country	Boys				Girls			
		11 Years	13 Years	15 Years	Total	11 Years	13 Years	15 Years	Total
2006	Estonia	688	728	801	2217	733	741	786	2260
	Latvia	715	691	628	2034	710	775	702	2187
	Lithuania	951	1013	940	2904	913	894	921	2728
	HBSC total (41 countries/regions)	32,832	34,394	33,007	100,233	33,875	35,560	34,865	104,300
2010	Estonia	669	692	661	2022	747	718	737	2202
	Latvia	713	675	666	2054	779	722	709	2210
	Lithuania	923	872	945	2740	888	848	847	2583
	HBSC total (40 countries/regions)	33,267	35,246	35,477	103,990	34,657	36,729	36,175	107,561
2014	Estonia	662	737	638	2037	692	691	631	2014
	Latvia	884	972	784	2640	970	983	942	2895
	Lithuania	1008	998	904	2910	1007	1019	794	2820
	HBSC total (42 countries/regions)	34,727	37,388	35,194	107,309	35,703	38,101	36,843	110,647
2018	Estonia	780	817	759	2356	779	788	783	2350
	Latvia	744	776	660	2180	791	743	682	2216
	Lithuania	665	667	572	1904	672	595	610	1877
	HBSC total (45 countries/regions)	38,213	39,031	35,089	112,333	39,007	39,248	36,853	115,108

**Table 2:** Prevalence of the health-related variables by country, survey year and gender (percentage and 95% confidence interval).

Variable	Year	Boys (11–15 Years)				Girls (11–15 Years)			
		Estonia	Latvia	Lithuania	HBSC Total	Estonia	Latvia	Lithuania	HBSC Total
Physical activity at least 60 min a day	2006	21.3 (19.6–23.0)	27.6 (25.6–29.6)	22.7 (21.2–24.2)	24.7 (24.4–25.0)	14.4 (13.0–15.9)	18.6 (17.0–20.2)	15.6 (14.2–17.0)	15.6 (15.4–15.8)
	2010	16.6 (15.0–18.2)	24.5 (22.6–26.4)	19.7 (18.2–21.2)	23.5 (23.2–23.8)	12.3 (10.9–13.7)	16.0 (14.5–17.6)	13.3 (12.0–14.6)	13.7 (13.5–13.9)
	2014	20.8 (19.0–22.6)	22.0 (20.4–23.6)	26.0 (24.4–27.6)	25.3 (25.0–25.6)	12.1 (10.7–13.5)	15.3 (14.0–16.6)	15.4 (14.1–16.7)	15.6 (15.4–15.8)
	2018	17.3 (15.8–18.8)	22.2 (20.5–24.0)	20.4 (18.6–22.2)	23.2 (22.9–23.5)	13.7 (12.3–15.1)	15.4 (13.9–16.9)	16.1 (14.4–17.8)	15.5 (15.3–15.7)
Consumption of vegetables daily	2006	19.0 (17.4–20.6)	19.0 (17.3–20.7)	22.4 (20.9–23.9)	28.2 (27.9–28.5)	23.0 (21.3–24.7)	27.5 (25.6–29.4)	26.8 (25.1–28.5)	35.3 (35.0–35.6)
	2010	18.7 (17.0–20.4)	21.6 (19.8–23.4)	22.7 (21.1–24.3)	29.4 (29.1–29.7)	21.3 (19.6–23.0)	27.8 (25.9–29.7)	30.9 (29.1–32.7)	36.8 (36.5–37.1)
	2014	22.3 (20.5–24.1)	21.4 (19.8–23.0)	26.4 (24.8–28.0)	31.7 (31.4–32.0)	25.8 (23.9–27.7)	29.6 (27.9–31.3)	34.8 (33.0–36.6)	39.0 (38.7–39.3)
	2018	29.0 (27.2–30.8)	24.3 (22.5–26.1)	29.7 (27.6–31.8)	34.9 (34.6–35.2)	36.0 (34.1–37.9)	30.1 (28.2–32.0)	38.8 (36.6–41.0)	42.0 (41.7–42.3)
Consumption of soft drinks daily	2006	12.9 (11.5–14.3)	13.5 (12.0–15.0)	16.4 (15.0–17.8)	27.3 (27.0–27.6)	6.9 (5.8–7.9)	12.0 (10.6–13.4)	13.0 (11.7–14.3)	22.3 (22.0–22.6)
	2010	8.2 (7.0–9.4)	9.7 (8.4–11.0)	10.6 (9.4–11.8)	23.5 (23.2–23.8)	4.6 (3.7–5.5)	7.0 (5.9–8.1)	5.9 (5.0–6.8)	18.4 (18.2–18.6)
	2014	8.5 (7.3–9.7)	7.9 (6.9–8.9)	14.7 (13.4–16.0)	19.7 (19.5–19.9)	4.6 (3.7–5.5)	5.1 (4.3–5.9)	8.3 (7.3–9.3)	15.4 (15.2–15.6)
	2018	7.4 (6.3–8.5)	8.5 (6.9–8.9)	16.2 (14.5–17.7)	17.5 (17.3–17.7)	3.7 (2.9–4.5)	4.0 (3.2–4.8)	9.0 (7.7–10.3)	13.3 (13.1–13.5)
Overweight or obese ^a^	2006	12.8 (11.4–14.2)	10.3 (8.9–11.7)	10.3 (8.9–11.7)	16.4 (16.1–16.7)	7.2 (6.1–8.3)	5.7 (4.7–6.7)	4.8 (3.8–5.7)	10.6 (10.4–10.8)
	2010	17.8 (15.9–19.7)	13.8 (12.2–15.4)	14.0 (12.4–15.6)	18.3 (18.0–18.6)	12.0 (10.5–13.5)	8.9 (7.7–10.1)	7.5 (6.3–8.7)	11.9 (11.7–12.1)
	2014	17.5 (15.7–19.3)	17.6 (16.1–19.1)	14.8 (13.3–16.4)	18.2 (17.9–18.5)	11.5 (10.0–13.0)	12.8 (11.6–14.0)	8.3 (7.0–9.5)	12.1 (11.9–12.3)
	2018	21.0 (19.2–22.8)	18.5 (16.8–20.2)	19.6 (17.6–21.5)	18.7 (18.4–19.0)	14.7 (13.2–16.2)	14.8 (13.3–16.3)	12.3 (10.7–13.8)	12.8 (12.6–13.0)

^a^ Missing values rates 2006–2018: Estonia 12.4%, Latvia 6.4%, Lithuania 27.4%, HBSC total 19.5%.

**Table 3:** Physical activity at least 60 min a day by survey year, age group and family affluence (odds ratios and 95% confidence intervals).

Variable	Category	Boys			Girls		
		Estonia	Latvia	Lithuania	Estonia	Latvia	Lithuania
Year ^a^	2006	1.00	1.00	1.00	1.00	1.00	1.00
	2010	0.73 (0.36–0.86) ***	0.85 (0.74–0.98) *	0.83 (0.73–0.95) **	0.83 (0.70–0.99) *	0.84 (0.71–0.98) *	0.83 (0.71–0.97) *
	2014	0.97 (0.83–1.12)	0.74 (0.64–0.84) ***	1.19 (1.06–1.35) **	0.82 (0.69–0.98) *	0.79 (0.68–0.92) **	0.98 (0.85–1.14)
	2018	0.77 (0.67–0.89) ***	0.75 (0.65–0.86) ***	0.87 (0.76–1.01)	0.94 (0.80–1.11)	0.80 (0.68–0.94) **	1.03 (0.88–1.21)
Year ^b^	2006	1.00	1.00	1.00	1.00	1.00	1.00
	2010	0.63 (0.54–0.74) ***	0.79 (0.68–0.91) **	0.79 (0.70–0.91) **	0.75 (0.63–0.90) **	0.82 (0.70–0.96) *	0.78 (0.67–0.92) **
	2014	0.87 (0.74–1.01)	0.69 (0.60–0.79) ***	1.08 (0.95–1.23)	0.74 (0.62–0.89) **	0.77 (0.66–0.89) **	0.87 (0.74–1.01)
	2018	0.63 (0.54–0.74) ***	0.66 (0.57–0.76) ***	0.81 (0.70–0.94) **	0.82 (0.69–0.98) *	0.73 (0.62–0.86) ***	0.98 (0.83–1.15)
Age ^b^	11 years	1.00	1.00	1.00	1.00	1.00	1.00
	13 years	0.92 (0.81–1.05)	0.84 (0.74–0.94) **	0.87 (0.78–0.97) *	0.62 (0.54–0.72) ***	0.72 (0.63–0.82) ***	0.62 (0.54–0.71) ***
	15 years	0.76 (0.66–0.87) ***	0.80 (0.71–0.91) **	0.73 (0.65–0.82) ***	0.50 (0.43–0.59) ***	0.65 (0.57–0.75) ***	0.57 (0.49–0.65) ***
FAS ^b,c^		1.11 (1.08–1.14) ***	1.13 (1.10–1.16) ***	1.08 (1.06–1.11) ***	1.08 (1.05–1.12) ***	1.07 (1.04–1.10) ***	1.06 (1.03–1.09) ***

^a^ Unadjusted OR-values; ^b^ Adjusted for age group and FAS; ^c^ Family affluence scale (FAS), added to regression model as a covariate; * *p* < 0.05; ** *p* < 0.01; *** *p* < 0.001.

**Table 4:** Consumption of vegetables daily by survey year, age group and family affluence (odds ratios and 95% confidence intervals).

Variable	Category	Boys			Girls		
		Estonia	Latvia	Lithuania	Estonia	Latvia	Lithuania
Year ^a^	2006	1.00	1.00	1.00	1.00	1.00	1.00
	2010	0.98 (0.84–1.14)	1.18 (1.01–1.37) *	1.02 (0.90–1.16)	0.91 (0.79–1.04)	1.01 (0.89–1.16)	1.22 (1.09–1.38) ***
	2014	1.22 (1.05–1.42) **	1.16 (1.00–1.34) *	1.24 (1.10–1.40) ***	1.16 (1.01–1.34) *	1.11 (0.98–1.26)	1.46 (1.30–1.63) ***
	2018	1.74 (1.51–2.00) ***	1.37 (1.18–1.58) ***	1.46 (1.28–1.67) ***	1.88 (1.65–2.14) ***	1.14 (1.00–1.30)	1.73 (1.53–1.97) ***
Year ^b^	2006	1.00	1.00	1.00	1.00	1.00	1.00
	2010	0.85 (0.73–1.00)	1.16 (0.99–1.36)	0.96 (0.85–1.10)	0.80 (0.69–0.93) **	0.99 (0.86–1.13)	1.14 (1.01–1.29) *
	2014	1.09 (0.93–1.27)	1.16 (1.00–1.34)	1.13 (1.00–1.28)	1.03 (0.89–1.19)	1.08 (0.96–1.23)	1.29 (1.14–1.45) ***
	2018	1.49 (1.29–1.72) ***	1.34 (1.15–1.56) ***	1.38 (1.21–1.58) ***	1.58 (1.38–1.81) ***	1.08 (0.95–1.24)	1.63 (1.43–1.85) ***
Age ^b^	11 years	1.00	1.00	1.00	1.00	1.00	1.00
	13 years	0.86 (0.76–0.97) *	0.82 (0.73–0.93) **	0.76 (0.68–0.85) ***	0.79 (0.70–0.89) ***	0.87 (0.78–0.97) *	0.73 (0.66–0.81) ***
	15 years	0.70 (0.61–0.79) ***	0.76 (0.67–0.86) ***	0.73 (0.65–0.82) ***	0.78 (0.69–0.88) ***	0.88 (0.79–0.99) *	0.70 (0.63–0.78) ***
FAS ^b,c^		1.11 (1.08–1.14) ***	1.02 (1.00–1.05)	1.07 (1.04–1.09) ***	1.12 (1.09–1.15) ***	1.05 (1.03–1.07) ***	1.08 (1.06–1.10) ***

^a^ Unadjusted OR-values; ^b^ Adjusted for age group and FAS; ^c^ Family affluence scale, added to regression model as a covariate; * *p* < 0.05; ** *p* < 0.01; *** *p* < 0.001.

**Table 5:** Consumption of soft drinks daily by survey year, age group and family affluence (odds ratios and 95% confidence intervals).

Variable	Category	Boys			Girls		
		Estonia	Latvia	Lithuania	Estonia	Latvia	Lithuania
Year ^a^	2006	1.00	1.00	1.00	1.00	1.00	1.00
	2010	0.61 (0.50–0.74) ***	0.69 (0.57–0.83) ***	0.60 (0.52–0,71) ***	0.65 (0.51–0.85) **	0.55 (0.45–0.68) ***	0.42 (0.34–0.51) ***
	2014	0.63 (0.51–0.76) ***	0.55 (0.46–0.67) ***	0.88 (0.76–1.01)	0.66 (0.50–0.85) **	0.39 (0.32–0.49) ***	0.60 (0.51–0.72) ***
	2018	0.54 (0.44–0.66) ***	0.59 (0.49–0.72) ***	0.98 (0.84–1.15)	0.52 (0.40–0.68) ***	0.30 (0.24–0.39) ***	0.66 (0.54–0.80) ***
Year ^b^	2006	1.00	1.00	1.00	1.00	1.00	1.00
	2010	0.54 (0.43–0.66) ***	0.67 (0.55–0.83) ***	0.59 (0.50–0.69) ***	0.67 (0.51–0.87) **	0.55 (0.45–0.68) ***	0.43 (0.35–0.52) ***
	2014	0.56 (0.46–0.69) ***	0.55 (0.45–0.67) ***	0.83 (0.71–0.96) *	0.66 (0.50–0.87) **	0.39 (0.32–0.49) ***	0.61 (0.51–0.74) ***
	2018	0.46 (0.37–0.57) ***	0.58 (0.47–0.71) ***	0.95 (0.81–1.11)	0.52 (0.39–0.70) ***	0.30 (0.23–0.38) ***	0.67 (0.55–0.82) ***
Age ^b^	11 years	1.00	1.00	1.00	1.00	1.00	1.00
	13 years	1.08 (0.90–1.29)	1.24 (1.04–1.48) *	0.93 (0.82–1.07)	0.95 (0.75–1.18)	1.19 (0.98–1.45)	1.15 (0.97–1.35)
	15 years	0.84 (0.70–1.02)	1.18 (0.98–1.41)	0.91 (0.79–1.04)	0.58 (0.45–0.75) ***	0.92 (0.75–1.13)	0.92 (0.77–1.09)
FAS ^b,c^		1.10 (1.06–1.15) ***	1.07 (1.04–1.11) ***	1.02 (1.00–1.15)	0.99 (0.94–1.04)	1.03 (0.99–1.08)	0.98 (0.95–1.01)

^a^ Unadjusted OR-values; ^b^ Adjusted for age group and FAS; ^c^ Family affluence scale, added to regression model as a covariate; * *p* < 0.05; ** *p* < 0.01; *** *p* < 0.001.

**Table 6:** Proportion of overweight/obese by survey year, age group and family affluence (odds ratios and 95% confidence intervals).

Variable	Category	Boys			Girls		
		Estonia	Latvia	Lithuania	Estonia	Latvia	Lithuania
Year ^a^	2006	1.00	1.00	1.00	1.00	1.00	1.00
	2010	1.48 (1.23–1.77) ***	1.39 (1.14–1.70) **	1.41 (1.16–1.72) ***	1.76 (1.42–2.19) ***	1.61 (1.26–2.05) ***	1.63 (1.24–2.13) ***
	2014	1.45 (1.21–1.73) ***	1.86 (1.55–2.23) ***	1.50 (1.24–1.82) ***	1.69 (1.36–2.10) ***	2.41 (1.94–3.00) ***	1.80 (1.38–2.35) ***
	2018	1.82 (1.54–2.15) ***	1.97 (1.63–2.38) ***	2.11 (1.74–2.56) ***	2.24 (1.82–2.74) ***	2.86 (2.29–3.58) ***	2.82 (2.18–3.63) ***
Year ^b^	2006	1.00	1.00	1.00	1.00	1.00	1.00
	2010	1.53 (1.27–1.85) ***	1.39 (1.13–1.71) **	1.42 (1.16–1.73) ***	1.87 (1.50–2.33) ***	1.60 (1.25–2.05) ***	1.70 (1.29–2.23) ***
	2014	1.54 (1.28–1.85) ***	1.84 (1.52–2.21) ***	1.51 (1.24–1.85) ***	1.76 (1.41–2.20) ***	2.44 (1.96–3.04) ***	1.91 (1.45–2.52) ***
	2018	1.97 (1.65–2.35) ***	1.98 (1.64–2.40) ***	2.11 (1.74–2.57) ***	2.43 (1.96–3.02) ***	2.87 (2.28–3.60) ***	2.85 (2.20–3.68) ***
Age ^b^	11 years	1.00	1.00	1.00	1.00	1.00	1.00
	13 years	0.94 (0.81–1.09)	0.93 (0.80–1.07)	0.78 (0.67–0.92) **	0.86 (0.72–1.01)	0.90 (0.77–1.05)	0.77 (0.63–0.94) **
	15 years	0.88 (0.75–1.02)	0.79 (0.68–0.92) **	0.70 (0.60–0.83) ***	0.64 (0.54–0.76) ***	0.67 (0.56–0.79) ***	0.60 (0.49–0.74) ***
FAS ^b,c^		0.95 (0.92–0.98) **	0.99 (0.96–1.02)	0.98 (0.95–1.02)	0.95 (0.91–0.98) **	0.99 (0.96–1.03)	0.94 (0.91–0.98) **

^a^ Unadjusted OR-values; ^b^ Adjusted for age group and FAS; ^c^ Family affluence scale, added to regression model as a covariate; ** *p* < 0.01; *** *p* < 0.001.
